# Bivalirudin in Patients Undergoing PCI: State of Art and Future Perspectives.

**Published:** 2016-05-16

**Authors:** G Galasso, M Mirra, G De Luca, F Piscione

**Affiliations:** 1 Department of Medicine and Surgery, University of Salerno, Salerno, Italy.; 2 Department of Translational Medical Sciences, University of Naples Federico II, Naples, Italy.; 3 Division of Cardiology, AOU Maggiore della Carità-Eastern Piedmont University, Novara, Italy.

**Keywords:** bivalirudin, anticoagulant, percutaneous coronary intervention, myocardial infarction, bleeding

## Abstract

Acute coronary syndrome (ACS) represents the most common cause of death worldwide. Percutaneous coronary intervention (PCI) is the management of choice in patients with ACS and occurrence of intra-procedural thrombotic complications are an independent predictor of mortality and other major adverse cardiovascular events in patients undergoing PCI. According to current guideline, anticoagulation therapy is indicated during PCI in order to reduce the risk of thrombotic complications such as stent thrombosis. Among currently available anticoagulant drugs, bivalirudin demonstrates a lower incidence of bleeding risk, despite it is associated with an increased risk of stent thrombosis. The aim of this paper is to discuss the pharmacology of bivalirudin and the clinical evidences of its use in patients undergoing PCI for ACS.

## I. INTRODUCTION

Coronary artery disease (CAD) represents the most common cause of death worldwide. According to characteristic electrocardiographic modifications, ACS are classified in: ST-segment elevation myocardial infarction (STEMI), non-ST-segment elevation myocardial infarction (NSTEMI) and unstable angina (UA) if cardiac biomarkers are negative^1^.

PCI is the management of choice in patients with ACS^2^,^3^. The occurrence of intraprocedural thrombotic complications is an independent predictor of cardiovascular mortality and major adverse cardiovascular events (MACE) in patients undergoing PCI^4^,^5^ and, according to current guideline, anticoagulation therapy is indicated, during PCI, in order to reduce this risk. Unfractionated heparin (UFH), low molecular weight heparin (LMWH) and fondaparinux, were considered the anticoagulants of choice for years, while, recently, bivalirudin, has been indicated in patients undergoing PCI^6^. The 2013 American College of Cardiology Foundation and American Heart Association guideline for management of patients with ST segment elevation myocardial infarction, recommends UFH with or without planned glycoprotein IIb/IIIa inhibitor (GPI) or bivalirudin as class I indication for patients undergoing primary PCI, with a preference for bivalirudin over UFH plus GPI in patients at high risk of bleeding (class IIa)^7^. The 2012 European Society of Cardiology guideline, however, recommend bivalirudin over UFH plus GPI (class I) but also LMWH (with or without GPI) over UFH (class IIb)^8^. Among currently available anticoagulant drugs, bivalirudin demonstrates a lower incidence of bleeding risk, despite it is associated with an increased risk of stent thrombosis. The aim of this paper is to discuss the pharmacology of bivalirudin and the clinical evidences of its use in patients undergoing PCI for an ACS.

## II. PHARMACOLOGY OF BIVALIRUDIN

**Figure 1.**
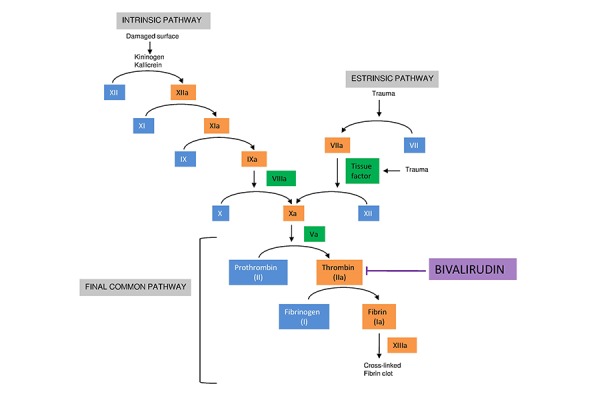
**The clotting cascade and role of bivalirudin.**

Bivalirudin is a transient and reversible thrombin inhibitor, preventing initiation and continuation of clot formation (Figure 1). It is a semi synthetic peptide of 20-amino acid peptide (2,180 Da molecular weight) derived from Irudin and extracted from Hirudo medicinalis^9^, with an half-life of 25 minutes and a volume of distribution of 0.24 l/kg; after intravenous administration it has a complete and immediate bioavailability. Bivalirudin has a lack of binding to plasma proteins and about 20% is excreted unmodified in the urine; renal failure prolongs its half-time up to four hours. No pharmacokinetic modifications have been observed in different age or gender^10^ (Table 1).

Currently, the recommended dose for patients undergoing PCI is a bolus of 0.75 mg/kg followed by an infusion of 1.75mg/kg/h for the duration of the procedure^6^. Unfortunately, no specific antidote exists for bivalirudin intoxication/overdosage and hemodialysis, hemofiltration, or plasmapheresis seem to be helpful in case of overdosage^11^.

## III. BIVALIRUDIN IN STEMI SETTING

Randomized trials testing the use of bivalirudin in STEMI setting are listed in Table 2.

The first study assessing bivalirudin safety and efficacy in STEMI setting was the Harmonizing Outcomes With Revascularization and Stents in Acute Myocardial Infarction (HORIZONS-AMI), performed in a large population of 3602 STEMI patient undergoing primary PCI^12^. The aim of this perspective, open-label, multicenter, randomized trial was to evaluate the incidence of major bleeding and combined adverse clinical events within 30 days of bivalirudin administration compared with UFH plus GPI, in STEMI patients. Combined adverse clinical events were defined as death, reinfarction, target-vessel revascularization for ischemia, and stroke. Patients were randomly assigned, in an open-label fashion and in a 1:1 ratio, to treatment with UFH plus GPI (the control group), both started before PCI, or to treatment with bivalirudin alone, administered by intravenous bolus of 0.75 mg/kg followed by an infusion of 1.75 mg/kg/hour discontinued at the completion of PCI. About two thirds of patients in the bivalirudin arm were pretreated with a UFH bolus before cardiac catheterization, and in 7.2% of them GPI were administered due to giant thrombus or no reflow after PCI. Patients in bivalirudin arm had a significantly reduced rate of net adverse clinical events (9.2 vs. 12.1%; relative risk [RR], 0.76; 95% confidence interval [CI], 0.63 to 0.92; p=0.005), due to a lower rate of major bleeding (4.9 vs. 8.3%; RR, 0.60; 95% CI, 0.46 to 0.77; p<0.001), with similar rates of MACE. Furthermore, they showed lower 30-days mortality (2.1 vs. 3.1%; RR, 0.66; 95% CI, 0.44 to 1.00; p=0.047), lower non-coronary artery bypass graft (CABG)-related bleedings (4.9 vs. 8.3%; p<0.001), while an increased risk of acute stent thrombosis was observed (1.3% vs. 0.3%, p<0.001). After 1 year, the rate of net adverse clinical events was lower in the bivalirudin group than in the control group (15.6% vs 18.3%, hazard ratio [HR] 0.83, 95% CI 0.71–0.97, p= 0.022), as a result of a lower rate of major bleeding in the bivalirudin group (5.8%vs 9.2%, HR 0.61, 0.48–0.78, p<0.0001). The rate of MACE was similar between groups (11.9%vs 11.9%, HR 1.00, 0.82–1.21, p=0.98). The 1-year rates of cardiac mortality (2.1% vs 3.8%, HR 0.57, 0.38–0.84, p=0.005) and all-cause mortality (3.5%vs 4.8%, HR 0.71, 0.51–0.98, p=0.037) were lower in the bivalirudin group than in the control group. The rate of stent thrombosis at 1 year was similar in both groups^13^. These findings were confirmed up to 3 years follow-up, when patients in bivalirudin arm showed lower rates of all-cause mortality (5.9 vs. 7.7%, difference −1.9% [−3.5 to −0.2], HR 0.75 [0.58–0.97]; p=0.03), cardiac mortality (2.9 vs. 5.1%, −2.2% [−3.5 to −0.9], 0.56 [0.40–0.80]; p= 0.001), reinfarction (6.2 vs. 8.2%, −1.9% [−3.7 to −0.2], 0.76 [0.59–0.99]; p=0.04), and major bleeding not related to bypass graft surgery (6.9 vs. 10.5%, −3.6% [−5.5 to −1.7], 0.64 [0.51–0.80]; p=0.0001) with no significant differences in ischemia-driven target vessel revascularisation, stent thrombosis, or composite adverse events^14^. Taken together these data indicate that bivalirudin use during PCI is safe with a significant reduction of ischemic adverse events but with an increased risk of acute stent thrombosis. A further randomized trial comparing bivalirudin, with UFH and routine use GPI, the European Ambulance Acute Coronary Syndrome Angiography (EUROMAX) study was performed. EUROMAX was a randomized, international, prospective, open-label ambulance trial comparing bivalirudin (0.75 mg/kg bolus followed immediately by a 1.75-mg/kg per hour infusion for ≥30 minutes prior to primary PCI and continued for ≥4 hours after the end of the procedure at the reduced dose of 0.25 mg/kg per hour) with UFH with or without GPI in 2200 patients with STEMI undergoing primary PCI. The primary end point was the evaluation of the incidence of death from any cause or non-CABG-related major bleeding at 30 days^15^. Patients in bivalirudin arm showed lower rate of death from any cause or non-CABG-related major bleeding (5.1 vs. 8.5%; RR 0.6; 95% CI 0.43–0.82; p=0.001). No significant differences in reinfarction rate and all cause mortality were observed in both groups. The incidence of stent thrombosis was higher in bivalirudin arm in the first 24 hours (1.1% vs. 0.2%; RR 6.11, 95% CI 1.37–27.24, p=0.007), being comparable after 24 hours up to 30 days. The composite outcome of net adverse clinical events (death, reinfarction, ischemia-driven revascularization, or stroke) incidence was lower in bivalirudin arm (7.8 vs. 10.6%; RR 0.73, 95% CI 0.56–0.96, p=0.02). This trial confirmed the reduced bleeding risk in patients treated with bivalirudin, but, despite a prolonged drug administration, the incidence of stent thrombosis was still higher in the first 24 hours despite the use of more potent thienopyridine agents. To test the hypothesis that association of bivalirudin plus GPI was superior to bivalirudin alone in reducing stent thrombosis, a trial comparing antithrombotic therapy with bivalirudin or UFH during PCI, How Effective are Antithrombotic Therapies in Primary Percutaneous Coronary Intervention (HEAT-PPCI) trial was then performed. HEAT-PPCI was an open-label, single centre, randomized trial, in which 1812 STEMI patients were randomized (1:1; stratified by age [<75 years vs ≥75 years] and presence of cardiogenic shock [yes vs no]) to UFH (70 U/kg) or bivalirudin (bolus 0.75 mg/kg; infusion 1.75 mg/kg/h for the duration of the procedure). Primary efficacy outcome, defined as a composite of all-cause mortality, cerebrovascular accident, reinfarction, or unplanned target lesion revascularisation, and primary safety, defined as incidence of major bleeding were evaluated at 28 days^16^. GPI was administered with no significant differences in both groups, in presence of massive thrombus, slow or no-reflow, or a thrombotic complication (13% in the bivalirudin and 15% in the UFH group). The primary efficacy outcome at 28 days was prevalent in bivalirudin arm (8.7 vs. 5.7%; RR 1.52, 95% CI 1.09–2.13, p=0.01). Bivalirudin patients showed an higher reinfarction and revascularization rate (2.7 vs. 0.9%, p=0.004; 2.7 vs. 0.7%, p=0.001, respectively). These findings may be explained with the abrupt discontinuation of bivalirudin infusion as soon as angioplasty was terminated, thus increasing the incidence of stent thrombosis (3.4 vs. 0.9%, p= 0.001). No significant differences were observed in both groups in the primary safety outcome at 28 days. In order to evaluate efficacy and safety of the association between bivalirudin and prasugrel, compared with UFH and clopidogrel the Bavarian Reperfusion Alternatives Evaluation (BRAVE) 4 study was performed. BRAVE 4 was a randomized, open-label, multicenter trial, comparing prasugrel plus bivalirudin to clopidogrel plus UFH in 548 STEMI patients planned for primary PCI^17^. Aim of this study was test the hypothesis that in STEMI patients undergoing primary PCI, prasugrel plus bivalirudin therapy is superior to clopidogrel plus heparin in terms of net clinical outcome (the composite of death, recurrent myocardial infarction, unplanned infarct-related artery revascularization, stroke, definite stent thrombosis, or major bleeding). No significant difference between two groups in the composite ischemic endpoint, bleeding events, cardiac mortality, or definite stent thrombosis, were observed and, due to slow recruitment, the trial was prematurely stopped. The Bivalirudin in Acute Myocardial Infarction vs Heparin and GPI Plus Heparin Trial (BRIGHT) was performed to determine if bivalirudin is superior to UFH with or without tirofiban during primary PCI. BRIGHT was a multicenter, open-label, randomized trial, involving 2194 patients with acute myocardial infarction undergoing primary PCI (1925 patients with STEMI and 269 patients with NSTEMI). Patients were randomly assigned to receive bivalirudin with a post-PCI infusion (n = 735), heparin alone (n = 729), or UFH plus tirofiban with a post-PCI infusion (n = 730)^18^. Aim of the study was to determine if bivalirudin was superior to UFH alone or plus tirofiban during primary PCI in term of composite of all-cause mortality, reinfarction, target vessel revascularization, stroke, and any bleeding events at 30 days. Net adverse clinical events in patients treated with bivalirudin, UFH and UFH plus tirofiban were 8.8%, 13.2% and 17%, respectively (RR for bivalirudin vs. UFH 0.67, 95% CI 0.50–0.90, p=0.008). This finding was largely due to a reduction in bleeding events with bivalirudin, while no significant differences in major adverse cardiac, cerebral events or stent thrombosis among three groups were observed.

Taken together these data demonstrated a lower incidence of bleeding with bivalirudin without any reduction on mortality. The Minimizing Adverse Haemorrhagic Events by TRansradial Access Site and Systemic Implementation of angioX (MATRIX) was a randomised, multicentre trial comparing transradial against transfemoral access in patients with ACS undergoing PCI. Patients were randomized (1:1) to radial or femoral access and to receive bivalirudin, stopped at the end of PCI or prolonged infusion for at least 4–6 hours, or UFH with provisional GPI^19^. The primary outcome was the composite of death, non-fatal myocardial infarction, or stroke, evaluated at 30 days. No significant differences were observed in patients treated with bivalirudin compared to patients treated with UFH (10.3 vs. 10.9%, p=0.45). Furthermore, patients in bivalirudin arm showed a lower rate of bleeding but an higher rate of stent thrombosis (1.4 vs. 2.5%, p=0.001; 1 vs. 0.6%, p=0.048, respectively). Interestingly, patients in bivalirudin arm showed lower rate of all cause mortality (1.7 vs. 2.3%, p=0.042). Thus, despite several randomized studies, the safety and efficacy of bivalirudin compared with UFH, with or without GPI, in patients with acute myocardial infarction undergoing PCI are still uncertain. Among all these randomized trials, bivalirudin showed a significant reduction of bleeding rate with an higher incidence of early stent thrombosis. However, when bivalirudin was administered up to 4 hours after the end of PCI, as in the BRIGHT trial, no differences in the incidence of early stent thrombosis were observed between bivalirudin and UFH arms, suggesting that a prolonged bivalirudin administration may significantly reduces the incidence of early stent thrombosis. Furthermore, as demonstrate in the EUROMAX trial, prolonging the low dose bivalirudin administration after PCI, reduces incidence of major bleeding, with a reduction in the overall mortality rate but an increased risk of stent thrombosis within the first 24 hrs post PCI. This finding is confirmed in our experience in more than 600 STEMI patients undergoing PCI and prolonged bivalirudin administration up to 40 hours after PCI (unpublished data).

## IV. BIVALIRUDIN IN NSTEMI SETTING

Randomized trials testing the use of bivalirudin in NSTEMI setting are listed in Table 3.

The Acute Catheterization and Urgent Intervention Triage Strategy (ACUITY) was a prospective, open-label trial, enrolling patients with moderate- or high-risk NSTE-ACS, with a prevalence of NSTEMI patients (59%)^20^. This study compared three different therapeutic strategies: UFH or enoxaparin plus GPI, bivalirudin plus GPI, or bivalirudin alone (with provisional use of GPI during PCI). All patients underwent coronary angiography within 72 h after randomization (median of 19.5–19.8 h). Patients on GPI had a further randomization in a two-by-two factorial design to receive the GPI either immediately after randomization (upstream) or to defer administration in the cath-lab at PCI starting. The endpoints of composite ischemia (death, myocardial infarction, or unplanned revascularisation for ischemia), major bleeding, and net clinical outcomes (combining composite ischemia or major bleeding), were evaluated at 30-day. There were no significant differences in the rate of primary or secondary outcomes evaluated at 30 days between patients who receiving bivalirudin plus GPI and patients treated by UFH plus GPI (composite ischemia rate: 9 vs. 8%, p= 0.16; major bleeding: 8 vs. 7%, p=0.32; net clinical outcomes: 15 vs. 13%, p=0.1). Rates of composite ischemia were similar in those who received bivalirudin alone and those who received UFH plus GPI (9 vs. 8%, p=0.45); however, patients who received bivalirudin showed a significantly reduction of major bleeding, compared to UFH plus GPI group (4 vs. 7%, p<0.0001, RR 0.52, 95% CI 0.40–0.66), resulting in a trend towards better 30-day net clinical outcomes (12 vs. 13%, p=0.057; 0.87, 0.75–1.00). Furthermore, this trial demonstrated that transradial intervention compared to femoral arterial access was able to reduce major bleeding complication. Similar results were observed at 1 year follow-up^21^. No differences were observed in the composite endpoint of ischemia or all cause mortality as well as in deferred selective use of GPI compared with routine upstream use. The Randomized Trial to Evaluate the Relative PROTECTion against Post-PCI Microvascular Dysfunction and Post-PCI Ischemia among Anti-Platelet and Anti-Thrombotic Agents–Thrombolysis In Myocardial Infarction-30 (PROTECT–TIMI-30) was performed to evaluate GPI inhibition with eptifibatide when administered with indirect thrombin inhibition as compared to monotherapy with direct thrombin inhibition with bivalirudin among patients with NSTEMI. The PROTECT TIMI-30 was a randomized trial enrolling patients presenting with NSTE-ACS who had at least one of this risk factors (diabetes mellitus, positive cardiac biomarkers, ST-segment deviation > 0.5 mm, TIMI risk score ≥ 3) and were anticipated to undergo PCI^22^. Patients were randomized in three groups: UFH plus eptifibatide, enoxaparin plus eptifibatide or bivalirudin with provisional eptifibatide. No significant difference were observed between bivalirudin and both eptifibatide arms in the composite of death, myocardial infarction, or ischemia on Holter through 48 h, or the rate of TIMI major bleeding. The combination of GPI and UFH has not been compared with bivalirudin in NSTEMI patients and the Intracoronary Stenting and Antithrombosis Research-Rapid Early Action for Coronary Treatment (ISAR – REACT) 4 was performed to evaluate which treatment is more effective to prevent thrombotic and bleeding complications. ISAR-REACT 4 was a double blind double-dummy trial comparing UFH plus abciximab with bivalirudin in patients undergoing PCI due to NSTEMI^23^. All patients received 325–500 mg of aspirin and 600 mg of clopidogrel. The primary outcome, a composite of death, large recurrent myocardial infarction, urgent target-vessel revascularization, or major bleeding within 30 days, was similar in two groups (10.9 vs. 11%, p=0.94; RR 0.99, 95% CI 0.74–1.32). Death, any recurrent myocardial infarction, or urgent target-vessel revascularization were similar in both groups (12.8 vs. 13.4%, p=0.76; RR 0.96; 95% CI, 0.74 to 1.25). Major bleeding was prevalent in the abciximab group (4.6 vs. 2.6%, p=0.02; RR 1.84; 95% CI, 1.10 to 3.07). This study showed that abciximab and unfractionated heparin, as compared with bivalirudin, failed to reduce the rate of the primary end point and increased the risk of bleeding among patients with non-ST-segment elevation myocardial infarction undergoing PCI. These findings were confirmed at 1 year follow-up^24^. To evaluate the optimal adjunctive anticoagulation regimen for PCI in patients presenting ACS initially treated with fondaparinux, the SWITCH III trial was performed. SWITCH III was an open-label pilot trial comparing UFH to bivalirudin in patients presenting with ACS initially treated with fondaparinux and a loading dose of 600 mg of clopidogrel^25^. During PCI, patients were randomized to either bivalirudin or UFH therapy in a 1:1 fashion. GPI were used in 3.9% patients of bivalirudin and 12.2% patients of UFH group. The number of ischemic or bleeding events was very low and no significant differences were detected between groups. Current data suggest that bivalirudin treatment, compared to others anticoagulant drugs, in NSTEMI patients underwent PCI results in bleeding reduction with no differences in the rate of death, myocardial infarction or recurrent ischemia.

## V. BIVALIRUDIN IN STABLE OR UNSTABLE ANGINA SETTING

Randomized trials testing the use of bivalirudin in the management of stable or unstable angina are listed in Table 4.

The HIRULOG was a double blind trial enrolling patients with unstable angina or post-infarction angina undergoing urgent PCI^26^. Patients were randomized in two groups receiving either heparin or bivalirudin immediately before angioplasty. The primary end point was in hospital death, myocardial infarction, abrupt vessel closure, or rapid clinical deterioration of cardiac origin. Patients receiving bivalirudin did not show a significant reduction of the incidence of the primary end point (11.4 vs. 12.2%, p=0.26) but a significant lower incidence of bleeding (3.8 vs. 9.8%, p<0.001). After this trial, bivalirudin was approved for use in Europe and the United States as an alternative to UFH for patients with unstable angina during PCI. To determine the efficacy of bivalirudin compared with UFH plus GPI at 6 months and 1 year, the Randomized Evaluation in PCI Linking Angiomax to Reduced Clinical Events (REPLACE)-2 trial was performed^27^. Patients were randomized to receive intravenously bivalirudin (0.75 mg/kg bolus, 1.75 mg/kg per hour for the duration of PCI), with provisional GPI, or to receive UFH (65 U/kg bolus) with planned GPI (abciximab or eptifibatide). Patients undergoing PCI due to acute myocardial infarction were excluded. The primary end point was defined as a composite of death, myocardial infarction, severe ischemia requiring surgical or percutaneous revascularization, or major bleeding at 30 days. No significant differences were observed between groups in death, myocardial infarction and repeat revascularization (1.4 vs. 1%; HR, 0.70; 95% CI, 0.43–1.14; p=0.15; 7.4 vs. 8.2%; HR, 1.12; 95% CI, 0.93–1.34; p=0.24; 11.4 vs. 12.1% HR, 1.06; 95% CI, 0.91–1.23; p=0.45; respectively). Patients in bivalirudin arm showed a significant reduction of major bleeding (2.4 vs. 4.1%, p<0.001). Long-term (6 and 12 months) clinical outcomes were similar in both groups. The Authors concluded that bivalirudin was non-inferior to UFH/GPI concerning the acute ischemic end points and was associated with a lower rate of major bleeding. To assess the superiority of bivalirudin compared with UFH in patients with stable or unstable angina who undergo PCI after pretreatment with clopidogrel the Intracoronary Stenting and Antithrombosis Research -Rapid Early Action for Coronary Treatment (ISAR – REACT) 3 study was performed. ISAR-REACT 3 was a multicenter double-blind randomized trial, designed to compare bivalirudin with UFH in patients undergoing PCI for stable or unstable angina^28^. The aim of this study was to compare bivalirudin with UFH in patients who had stable or unstable angina pectoris undergoing PCI and stenting after pretreatment with 600 mg of clopidogrel and aspirin (325 to 500 mg). The primary end point was the composite of death, myocardial infarction, urgent target-vessel revascularization due to myocardial ischemia within 30 days after randomization, or major bleeding during the index hospitalization (with a net clinical benefit defined as a reduction in the incidence of the end point). The secondary end point was the composite of death, myocardial infarction, or urgent target-vessel revascularization. No significant differences were observed between groups in primary and secondary end point (8.3 vs. 8.7%; RR, 0.94; 95% CI, 0.77 to 1.15; p=0.57; 5.9 vs. 5.0%; RR 1.16; 95% CI, 0.91 to 1.49; p=0.23). The incidence of major bleeding was prevalent in UFH group (3.1 vs. 4.6%; RR, 0.66; 95% CI, 0.49 to 0.90; p=0.008). In conclusion, in patients with stable and unstable angina undergoing PCI after preloading with clopidogrel, bivalirudin did not provide a net clinical benefit, but significantly reduced the incidence of major bleeding. The Novel Approaches for Preventing or Limiting EventS (NAPLES) trial was performed to evaluate bivalirudin safety and efficacy in the high risk subset of diabetic patients undergoing PCI. NAPLES was a single centre randomized trial focusing on the management of diabetic patients undergoing elective PCI pretreated with a loading dose of clopidogrel of 300 mg^29^. Patients were randomized to receive bivalirudin monotherapy or UFH plus routine tirofiban. The primary composite end point (30-day composite incidence of death, urgent repeat revascularization, myocardial infarction, and all bleedings) was lower in the bivalirudin group (18.0% vs. 31.5%, OR 0.47, 95% CI 0.28 to 0.79, p=0.004). No death, urgent revascularization, or Q-wave myocardial infarction were observed. The rate of non-Q-wave myocardial infarction was similar in the 2 groups (10.2 vs. 12.5%, p=0.606). A lower rate of any bleeding was observed in bivalirudin arm (8.4 vs. 20.8%, OR 0.34, 95% CI 0.18 to 0.67, p=0.002). This difference was mainly ascribed to the lower rate of minor bleeding (7.8 vs. 18.5%, OR 0.37, 95% CI 0.19 to 0.74, p=0.005), although the rate of major bleeding in the 2 groups was similar (0.6 vs. 2.4%, p=0.371). In conclusion, in patients with diabetes undergoing elective PCI, bivalirudin allows a reduction of in-hospital bleeding. To evaluate the effectiveness of bivalirudin versus UFH in selected PCI patients at high bleeding risk the Anti-Thrombotic Strategy for Reduction of Myocardial Damage During Angioplasty-Bivalirudin vs Heparin (ARMYDA-7 BIVALVE) was performed. ARMYDA-7 BIVALVE was a randomized open-label trial in patients at high bleeding, due to age >75 years, chronic renal failure, and diabetes mellitus, with documented coronary artery disease undergoing PCI risk^30^. Patients were randomized to bivalirudin (bolus 0.75 mg/kg followed by infusion during the procedure; n = 198) or UFH (75 IU/kg; n = 203), with provisional use of GPI during PCI. Patients undergoing primary PCI for STEMI were excluded. The primary efficacy and safety end point were the 30-day incidence of major adverse cardiac events (cardiac death, myocardial infarction, stent thrombosis, or target vessel revascularization), and the occurrence of any bleeding or entry-site complications after PCI, respectively. All patients were preloaded with clopidogrel 600 mg. No statistically significant differences between two groups were observed in major adverse cardiac event rate at 30 days (11.1 vs. 8.9%, p=0.56). The occurrence of the primary safety end point was lower in bivalirudin group (1.5 vs. 9.9%; p= 0.0001) and, this benefit, was essentially driven by the prevention of entry-site hematomas >10cm (0.5% vs. 6.9%, p=0.002). In conclusion, bivalirudin induces significantly lower bleeding and has a similar incidence of major adverse cardiac events in elderly patients, in patients with diabetes mellitus, or chronic renal failure undergoing PCI. Novel Approaches in Preventing and Limiting Events III Trial: Bivalirudin in High-Risk Bleeding Patients (NAPLES) III study assessed the safety and the efficacy of bivalirudin compared with UFH alone in the subset of patients at increased risk of bleeding undergoing elective PCI by femoral access. NAPLES III was a single-center double-blind trial comparing UFH to bivalirudin in patients undergoing elective PCI who had negative biomarkers but an high bleeding risk^31^. The primary endpoint was the rate of in-hospital major bleeding and no significant differences were observed among two groups (2.6 vs. 3.3%; p=0.54). The results of this randomized study suggested that there was no difference in major bleeding rate between bivalirudin and UFH in increased-risk patients undergoing transfemoral PCI.

As suggested by latest ESC guideline on myocardial revascularization among PCI patients with negative biomarkers, bivalirudin reduced bleeding without affecting mortality and might therefore be considered for use in patients at high risk for bleeding^32^.

## VI. META-ANALYSES

Several meta-analyses have been recently published in order to clarify the role of bivalirudin for patients undergoing PCI in different setting of patients. Cavender and Sabatine performed a meta-analysis of 16 studies enrolling patients for planned PCI and randomly assigned to bivalirudin or heparin (UFH or low-molecular weight heparin) with or without a GPI^33^. The primary efficacy endpoint was the incidence of major adverse cardiac events up to 30 days. Secondary efficacy endpoints were death, myocardial infarction, ischemia-driven revascularisation, and stent thrombosis. The primary safety endpoint was major bleeding up to 30 days. Bivalirudin use was associated with an high risk of major adverse cardiac events (RR 1.09, 95% CI 1.01–1.17; p=0.0204), driven by an increases in myocardial infarction (1.12, 1.03–1.23) and ischaemia-driven revascularisation (1.16, 0.997–1.34) as compared with heparin, with no effect on mortality (0.99, 0.82–1.18). Furthermore, bivalirudin patients showed an increased risk of stent thrombosis (RR 1.38, 95% CI 1.09–1.74; p=0.0074), which was primarily due to an increase in acute cases in ST-segment elevation myocardial infarction (4.27, 2.28–8.00; p<0.0001). Moreover, bivalirudin patients showed lower major bleeding risk (RR 0.62, 95% CI 0.49–0.78; p<0.0001), but the magnitude of this effect varied greatly (p<0.0001) depending on whether GPI were used predominantly in the UFH arm only (0.53, 0.47–0.61; p<0.0001), provisionally in both arms (0.78, 0.51–1.19; p=0.25), or planned in both arms (1.07, 0.87–1.31; p= 0.53). In conclusion, the use of bivalirudin increases the risk of myocardial infarction and stent thrombosis, but decreases the risk of bleeding, with the magnitude of the reduction depending on concomitant GPI use. Current recommendations on the use of bivalirudin in patients treated with PCI are mostly based on trials comparing bivalirudin versus UFH plus planned GPI.

Whether bivalirudin is also superior to UFH alone is still not well established. A meta-analysis of Cassese et al. investigates the efficacy and safety of bivalirudin versus UFH in patients treated with PCI without planned use of GPI^34^. The primary efficacy and safety outcomes were the 30-day incidence of death and major bleeding, respectively. The secondary outcomes were the 30-day incidence of myocardial infarction, definite stent thrombosis, urgent target vessel revascularization, and overall death at the longest available follow-up. Odds ratio (OR) and 95%CI served as summary statistics. At 30 days, bivalirudin versus UFH showed a comparable risk of death (1.09 [0.83–1.41], p=0.54), and myocardial infarction (1.10 [0.83–1.46], p=0.50) with a trend towards a higher risk of urgent target vessel revascularization (1.37 [0.96–1.96], p=0.08). The risk of major bleeding was lower with bivalirudin (0.57 [0.40–0.80], p=0.001) and the bleeding reduction was more evident when high doses of UFH were used as comparator (p for interaction <0.001). The risk of definite stent thrombosis (2.09 [1.26–3.47], p=0.005) and, in particular, the risk of acute stent thrombosis (3.48 [1.66–7.28], p< 0.001) was increased by bivalirudin. In conclusion, patients undergoing PCI randomised to therapy with either bivalirudin or UFH showed a similar mortality. Bivalirudin appears to reduce the risk of major bleeding at the expense of a higher risk of acute stent thrombosis. The evaluation of early stent thrombosis with bivalirudin in patients undergoing PCI still unclear. Piccolo et al. performed a meta-analysis of randomized trials assessing the risk of early ST associated with bivalirudin as compared to other antithrombotic regimens in patients undergoing PCI^35^. Compared to other regimens, bivalirudin significantly increased the risk of early stent thrombosis (OR= 1.80; 95 % CI, 1.28–2.52; p=0.0007) and reduced the risk of major bleeding (OR [95 %CI]=0.64 [0.51–0.82], p=0.0003), with a comparable risk of mortality or myocardial infarction. The higher risk of early stent thrombosis was mainly due to acute (OR [95 % CI] =4.33 [2.33–8.05], p<0.001) rather than subacute (OR [95 % CI] =0.89 [0.53–1.50], p=0.67) ST events (p for interaction < 0.001). Non-fatal myocardial infarction was the most common presentation (83 %) of early stent thrombosis events, while death occurred infrequently (about 5 %). In conclusion, in patients undergoing PCI, bivalirudin compared to UFH is associated with a higher risk of early stent thrombosis, which is mainly related to more frequent acute events. Further studies are required to evaluate alternative strategies to mitigate this risk, without hampering the benefits derived from the reduction in bleeding events with bivalirudin. These meta-analyses substantially confirmed a reduction of bleeding, without a significant reduction of mortality rate in patients treated with bivalirudin; however a re-evaluation of current data adding MATRIX study patients demonstrate for the first time highly significant benefits of radial access in acute coronary syndrome patients for major adverse cardiovascular events (p= 0.0051) and all-cause mortality (p= 0.0011) in patients treated with bivalirudin^19^. In a recent meta-analysis, performed by Bavry AA et al. patients treated with bivalirudin, compared with those treated with UFH, showed a reduction of bleedings rate, but this difference was less significative when UFH dosage during PCI was reduced, or, when ACT control in the cathlab was performed for both study groups (UFH and Bivalirudin)^35^. Finally, as proposed by Navarese et al, comparing several anticoagulant regimen, a different approach based on an individualized hemorrhagic or ischemic risk assessment should be evaluate, performing a tailored therapy^36^.

## VII. CONCLUSIONS

It is widely demonstrated that the use of bivalirudin in patients undergoing PCI due to acute coronary syndrome or stable angina significantly reduces incidence of major bleeding; however it is associated with a higher risk of early stent thrombosis, which is mainly related to more frequent acute events. MATRIX results, for the first time, demonstrated that death for all causes was reduced in patients treated with bivalirudin after a radial access. BRIGHT trial demonstrated that a prolonged bivalirudin administration significantly reduced the incidence of the early stent thrombosis. Further randomized clinical trials are needed to evaluate the safety and efficacy of bivalirudin in the era of PCI through radial artery with the use of new P2Y12 inhibitors and bioadsorbable scaffold even in high bleeding risk patients in which bivalirudin seems to be safe as reported in some case report^37^. Future approaches should be based on patient risk stratification in order to choose the best therapeutic option in each case with particular regard to the prevention of stent thrombosis. Finally, bivalirudin has higher cost compared to unfractionated heparin and it should be considered in the actual scenario of limited financial resources.

## Figures and Tables

**Table 1.** Property of bivalirudin.

Mechanism of action	Direct inhibitor of thrombin
Effect on clot-bound thrombin	Inactivation
Administration	Intravenous
Molecular weight	2,180 Da
Half-life	25 minutes
Plasma proteins binding	No
Renal excretion	20%
Antidote	No
Age and gender pharmacokinetic modifications	No
Recommended dose	Bolus of 0.75 mg/kg followed by infusion of 1.7 5mg/kg/h for the duration of the procedure

**Table 2.** Summary on trials for bivalirudin in STEMI setting.

STUDY	YEAR	PATIENTS	INDICATION	GROUPS	OUTCOMES	EFFICACY	MAJOR BLEEDINGS
HORIZONS-AMI^12^–^14^	2008	3602	STEMI	UHF + GPI vs. bivalirudin	Death, reinfraction, stroke, revascularization or major bleeding at 30 days; Death, reinfraction, stroke, revascularization or major bleeding at 1 year; Death, reinfarction, stroke, revascularization or major bleeding 3 years.	12.1 vs 9.2% (p=0.005)	8.3 vs 4.9 (p<0.001)
EUROMAZ^15^	2013	2193	STEMI	UFH vs. bivalirudin	Death or major bleeding at 30 days.	8.5 vs 5.1% (p=0.001)	6 vs 2.6% (p=0.001)
HEAT-PPCI^16^	2014	1812	STEMI	UFH vs. bivalirudin	Death, stroke, reinfraction, or revascularization at 28 days.	5.7 vs 8.7% (p=0.01)	3.1 vs 3.5% (p=0.59)
BRAVE 4^17^	2014	548	STEMI	UFH +clopidogrel vs. bivalirudin+prasugrel	Death, MI, revascularization, stent thrombosis, stroke or bleeding at 30 days.	14.5 vs 15.6% (p=0.68)	2.9 vs 2.6% (p=0.97)
BRIGHT^18^	2015	2194	STEMI and NSTEMI	UFH vs. UFH+tirofiban vs. bivalirudin	Death, MI, revascularization, stroke or bleeding at 30 days	13.2 vs 17 vs 8.8% (p=0.008^*^ and p<0.001^**^)	7.5 vs 12.3 vs 4.1% (p<0.001^*^, p<0.001^**^)
MATRIX^19^	2015	8405	ACS	UFH vs. bivalirudin vs prolonged bivalirudin	Death, non-fatal MI or stroke at 30 days	10.9 vs 10.3^§^% (p=0.45)	2.5 vs 1.4^§^% (p=0.001)

STEMI: ST-elevation myocardial infarction; NSTEMI. non- ST-elevation myocardial infarction; ACS: acute coronary syndrome; UFH: unfractionated heparin; GPI: glycoprotein IIa/IIIa inhibitors; MI: myocardial infarction;

§ combinated outcome for short and long term bivalirudin administration;

* p for the comparison of UFH vs bivalirudin;

** p for the comparison of GPI vs bivalirudin.

**Table 3.** Summary on trials for bivalirudin in NSTEMI setting.

STUDY	YEAR	PATIENTS	INDICATION	GROUPS	OUTCOMES	EFFICACY	MAJOR BLEEDINGS
ACUITY^20^	2006	13819	Moderate or high risk NSTE-ACS	UFH/enoxaparin plus GPI vs. bivalirudin	Death, MI, or unplanned revascularization at 30 days	7.3 vs. 7.7 vs. 7.8% (p=0.39^*^ and p=0.32^**^)	5.7 vs. 5.3 vs. 3% (p= 0.38^*^ and p< 0.001^**^)
PROTECT TIMI-30^22^	2006	797	NSTE-ACS with ≥ 1 risk factor	Eptifibatide (with UFH or enoxaparin) vs. bivalirudin	Death, MI, or ischemia on Holter through 48 h	14.2 vs. 18% (p=0.15)	0.7 vs. 0% (p=0.308)
ISAR-REACT4^23^	2011	1721	NSTEMI	UFH plus abciximab vs. bivalirudin	Death, large MI, urgent revascularization or major bleeding	10.9 vs. 11% (p=0.94)	4.6 vs. 2.6% (p= 0.02)
SWITCH III^25^	2013	100	NSTE-ACS undergoing PCI	UFH vs. bivalirudin	–	–	0 vs. 2% (p=0.49)

NSTE-ACS: non-ST-elevation acute coronary syndrome; NSTEMI: non- ST-elevation myocardial infarction; PCI: percutaneoud coronary intervention; UFH: unfractionated heparin: GPI: glycoprotein IIb/IIIa inhibitors; MI: myocardial infarction;

* p for the comparison of UFH/enoxaparin plus GPI vs bivalirudin plus GPI;

** p for the comparison of UFH/enoxaparin plus GPI vs bivalirudin alone

**Table 4.** Summary on trials for bivalirudin in stable or unstable angina setting.

STUDY	YEAR	PATIENTS	INDICATION	GROUPS	OUTCOMES	EFFICACY	MAJOR BLEEDINGS
HIRULOG^26^	1995	4098	Unstable or postinfarction angina	Bivalirudin vs. UFH	Death, MI, repeated angioplasty, IABC or CABG during hospitaiization	11.8 vs. 12.9% (p=0.26)	3.8 vs. 9.8% (p< 0.001)
REPLACE-2^27^	2003	5966	Urgent or elective PCI	Bivalirudin & provisional GPI vs. UFH & planned GPI	Death, MI, ischemia requiring revascularization, major bleeding at 30 days	9.2 vs. 10% (p=0.32)	2.4 vs. 4.1% (p< 0.001)
ISAR-REACT 3^28^	2008	4570	Stable or unstable angina	Bivalirudin vs. UFH	Death, MI, revascularization at 30 days or in-hospital major bleeding	8.3 vs. 8.7% (p=0.57)	3.1 vs. 4.6% (p...
NAPLES^29^	2009	335	Elective PCI in diabetes	Bivalirudin vs. UFH plus tirofiban	Death, MI, urgent revascularization at 30 days .days or in-hospital bleeding	18 vs. 31% (p=0.004)	0.6 vs. 2.4 (p=0.37)
ARMYDA-7 BIVALVE^30^	2012	401	NSTE-ACS or stable angina	Bivalirudin vs. UFH	Cardiac death, MI, revascularization or stent thrombosis at 30 days	11.1 vs. 8.9% (p=0.56)	0.5 vs. 1% (p=0.98)
NAPLES III^31^	2015	837	Stable or unstable angina	Bivalirudin vs. UFH	Death, MI, revascularization, stent thrombosis or major bleeding at 30 days	6.5 vs. 4.3% (p= 0.17)	3.3 vs. 2.6% (p=0.54)

NSTE-ACS: non-ST-elevation acute coronary syndrome; CAD: coronary artery disease; UFH: unfractionated heparin; MI: myocardial infarction; IABC: intra-aortic balloon counterpulsation; CABG: coronary artery bypass grafting; GPI: glycoprotein IIb/IIIa inhibitors.
